# Tissue-specific roles of IGFBP2 in glucose and lipid metabolism in obesity-related metabolic diseases

**DOI:** 10.3389/fnut.2026.1814752

**Published:** 2026-04-21

**Authors:** Xiaoyu Song, Wentao Zhong, Hao Zheng, Xinyang Ou, Dongjin Zheng, Zhuo Li

**Affiliations:** Department of Endocrinology and Metabolism, The First Hospital of Jilin University, Changchun, Jilin, China

**Keywords:** IGFBP2, MASLD, metabolism, obesity, T2DM

## Abstract

Obesity-related metabolic diseases are characterized by profound disturbances in glucose and lipid metabolism across multiple organs, yet the mediators that coordinate these tissue-specific alterations remain incompletely understood. Insulin-like growth factor-binding protein 2 (IGFBP2), a circulating and locally expressed regulatory protein, has emerged as a context-dependent modulator of metabolic homeostasis with potential relevance to obesity, insulin resistance, type 2 diabetes mellitus (T2DM), and metabolic dysfunction-associated steatotic liver disease (MASLD). In this review, we integrate evidence from *in vitro* studies, animal models, and human investigations to examine the tissue-specific roles of IGFBP2 in the liver, adipose tissue, pancreas, skeletal muscle, and cardiovascular system. We further discuss IGFBP2 within an autocrine, paracrine, and endocrine framework, with emphasis on its effects on glucose handling, lipid metabolism, insulin sensitivity, and metabolic adaptation in obesity-related disease states. In addition, we summarize current clinical evidence supporting circulating IGFBP2 as a candidate biomarker of metabolic dysfunction and discuss how nutritional factors and metabolic interventions may influence its expression and circulating levels. Collectively, available evidence suggests that IGFBP2 is a context-dependent regulator and potential translational indicator of metabolic dysregulation; however, important gaps remain regarding its tissue-specific sources and modes of action, receptor interactions, context-specific signaling mechanisms, and the strength of prospective human evidence.

## 1 Introduction

The global prevalence of obesity, type 2 diabetes mellitus (T2DM), and metabolic dysfunction-associated steatotic liver disease (MASLD) continues to rise, placing an increasing burden on public health systems worldwide (1–5). According to the World Health Organization, more than 1 billion people were living with obesity in 2022, meaning that approximately 1 in 8 people worldwide were affected (6). Meanwhile, T2DM currently affects 588.7 million people and is projected to reach 852.5 million by 2050 (7). The global prevalence of non-alcoholic fatty liver disease (NAFLD), now included within the broader concept of MASLD, is approximately 24% (8). In this review, we use the current term “MASLD”; however, when cited studies used the historical term “NAFLD,” that terminology is retained only when directly referring to those earlier reports (9).

These conditions often cluster as metabolic syndrome (10, 11), accelerating end-organ damage. Obesity-induced insulin resistance (IR) disrupts lipid metabolism and sustains chronic inflammation (12, 13), driving the progression of MASLD to metabolic dysfunction-associated steatohepatitis (MASH), cirrhosis, and hepatocellular carcinoma (HCC) (14). Notably, T2DM is an independent risk factor for the development of NAFLD (15), and a history of gestational diabetes mellitus (GDM) also confers a higher risk of NAFLD compared to the general population (16, 17). Moreover, a diagnosis of NAFLD doubles the risk of developing T2DM (18, 19).

A hallmark of obesity-related metabolic diseases is the disruption of substrate metabolism, particularly glucose and lipid metabolism across multiple organs (20–22). In obesity, adipose tissue dysfunction and insulin resistance impair glucose disposal in peripheral tissues and are accompanied by increased hepatic glucose production (20, 21, 23). At the same time, excessive lipid flux and reduced adipose buffering capacity promote ectopic lipid deposition in the liver and skeletal muscle, contributing to lipotoxic stress, mitochondrial dysfunction, and progressive metabolic deterioration (20, 23–25).

Insulin-like growth factor-binding protein 2 (IGFBP2) is a secreted member of the insulin-like growth factor-binding protein family and is best viewed as a circulating as well as locally acting metabolic regulatory protein rather than a classical cytokine. The liver is considered an important contributor to circulating IGFBP2, whereas adipose tissue and skeletal muscle can also contribute to its local or context-dependent expression (26–28). IGFBP2 expression is sensitive to physiological and pathological cues, including fasting, nutritional state, leptin signaling, adiposity, and insulin sensitivity (27–29). Functionally, IGFBP2 influences metabolism through both IGF-dependent and IGF-independent mechanisms. At the local level, it has been implicated in adipogenesis, adipocyte glucose uptake, skeletal-muscle insulin responsiveness, and hepatic lipid handling (28, 30–32). At the systemic level, lower circulating IGFBP2 has been associated with obesity, adverse metabolic traits, and reduced insulin sensitivity in human studies. Prospective studies further suggest a link between lower IGFBP2 levels and future dysglycemia or type 2 diabetes (33–35). Structurally, IGFBP2 contains an integrin-binding Arg-Gly-Asp (RGD) motif and a heparin-binding domain (HBD), while its N- and C-terminal domains mediate interactions with insulin-like growth factor 1 (IGF-1) and IGF-2 (36, 37). The RGD motif facilitates binding to integrins such as αVβ3 and α5β1, whereas the HBD supports interactions with extracellular matrix components (38–40). Against this background, a comprehensive synthesis of the tissue-specific mechanisms by which IGFBP2 modulates glucose and lipid homeostasis across key metabolic organs remains lacking.

## 2 Effects of IGFBP2 on glucose and lipid metabolism

In addition to its metabolic roles, IGFBP2 is regulated by multiple mechanisms, including epigenetic modification, post-translational control, which may influence its expression and function. The expression of IGFBP2 can be modulated through various epigenetic mechanisms, including DNA methylation, histone acetylation, and non-coding RNAs (41–44). Moreover, the IGFBP2 protein itself can undergo functional modifications such as phosphorylation after synthesis (45, 46), altering how it interacts with IGF molecules and cell surface receptors and thereby influencing its metabolic roles (47). These regulatory layers may help explain why IGFBP2 expression is frequently altered under conditions of metabolic stress, such as obesity, insulin resistance, and steatotic liver disease. In the following sections, we therefore focus on evidence from rodent models and human studies to examine how changes in IGFBP2 relate to metabolic phenotypes and through which pathways these effects are thought to occur.

### 2.1 Effects of IGFBP2 on metabolism in rodents

Systemic overexpression of IGFBP2 in mice reduced fasting insulin levels and improved glucose tolerance, whereas *IGFBP2^–/–^* mice exhibited impaired glucose tolerance and insulin sensitivity (28, 48). In aged mice, IGFBP2 overexpression also protected against age-related insulin resistance, maintaining normal insulin levels under both fasting and fed conditions (49). Mechanistically, recombinant IGFBP2 directly inhibited 3T3-L1 adipocyte differentiation *in vitro*, suggesting that its anti-obesity effects may partly result from suppression of adipogenesis rather than solely from secondary systemic changes (49). Consistent with this, IGFBP2-overexpressing mice challenged with a high-fat diet showed reduced weight gain, lower abdominal fat mass, and smaller adipocytes (49). In addition, IGFBP2 was identified as a leptin-regulated gene, and adenoviral IGFBP2 overexpression markedly improved glucose metabolism in diabetic mouse models, with hyperinsulinemic clamp studies demonstrating a nearly threefold increase in hepatic insulin sensitivity in ob/ob mice (48).

In a rat model of GDM, microRNA-regulated IGFBP2 expression ameliorated insulin resistance and attenuated myocardial injury, further supporting a protective role for IGFBP2 under metabolic stress, although the underlying molecular mechanism was not fully defined (50). The regulation of IGFBP2 is also subject to epigenetic control. Fibroblast growth factor 1 (FGF1) was shown to reverse obesity-associated hypermethylation of the IGFBP2 promoter, concomitant with improvements in MASLD (51). Mechanistically, FGF1 restored IGFBP2 expression by reducing DNMT3A recruitment to its promoter, thereby decreasing promoter methylation; suppression of IGFBP2 blunted the beneficial effects of FGF1 on fasting glucose, insulin resistance, hepatic lipid accumulation, and lipogenic gene expression (51). In addition, Zhai et al. demonstrated that IGFBP2-knockout mice developed more severe hepatic steatosis after HFD feeding, with increased hepatic and circulating triglycerides, total cholesterol, and free fatty acids (32). Mechanistically, IGFBP2 bound EGFR and suppressed downstream EGFR–STAT3 signaling, whereas disruption of the IGFBP2–EGFR complex activated STAT3 and enhanced Srebf1 promoter activity, thereby promoting hepatic lipogenesis and steatosis (32). These findings indicate that, beyond its classical IGF-binding properties, IGFBP2 can regulate hepatic lipid metabolism through an IGF-independent receptor-mediated mechanism. Overall, current rodent evidence suggests that IGFBP2 exerts metabolically protective effects through suppression of adipogenesis and regulation of hepatic insulin sensitivity and lipogenesis, although mechanistic evidence outside the liver remains comparatively limited.

### 2.2 Effects of IGFBP2 on metabolism in humans

Clinical evidence indicates that circulating IGF-1 and IGF-2 are closely associated with insulin sensitivity and glucose tolerance (52, 53). Population-based studies have shown that individuals with low circulating IGF-1 levels exhibit an increased risk of developing T2DM (54, 55), while reduced IGF-2 levels are often associated with weight gain and obesity (56). As a classical IGF-binding protein, IGFBP2 regulates the bioavailability of IGF-1 and IGF-2 through high-affinity binding (37). The resulting complexes prolong the half-life of IGFs but transiently inhibit their biological activity, thereby preventing excessive signaling and hypoglycemia (57). Beyond its IGF-dependent functions, however, IGFBP2 also appears to reflect broader metabolic status, and numerous studies have reported significant correlations between circulating IGFBP2 levels and disorders of glucose and lipid metabolism (58–61).

**FIGURE 1:**
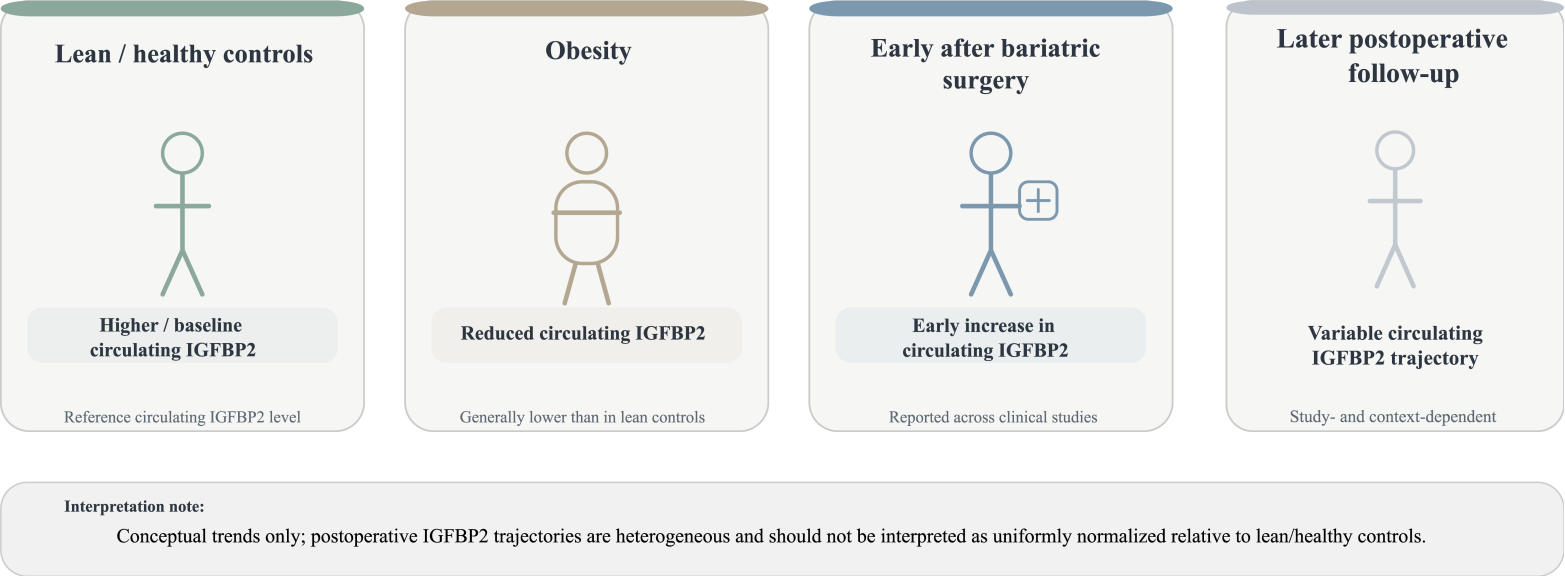
Conceptual summary of reported clinical trends in circulating IGFBP2 across lean/healthy controls, obesity, and post-bariatric states. Lean/healthy controls are shown as the reference state. Clinical studies generally report lower circulating IGFBP2 in obesity and an early increase after bariatric surgery, whereas later postoperative trajectories appear more variable across studies. This figure summarizes reported trends conceptually rather than quantitatively. Because of heterogeneity in study design, bariatric procedure, follow-up duration, and baseline metabolic characteristics, postoperative IGFBP2 levels should not be interpreted as uniformly normalized relative to lean/healthy controls. Based on references (64–66, 76).

Wittenbecher et al. found that higher IGFBP2 levels were associated with a lower risk of T2DM, whereas increased DNA methylation of the IGFBP2 gene was correlated with elevated T2DM risk (34). In obese children, serum IGFBP2 concentrations are decreased and inversely correlated with insulin sensitivity (62). A prospective study of 625 healthy adults aged 25–70 years over 6.2 years revealed that higher baseline serum IGFBP2 levels predicted a lower incidence of obesity and improved glucose tolerance (63). Notably, IGFBP2 appears to play a protective role against obesity. Although obese patients exhibit reduced serum IGFBP2 levels, bariatric surgery has been reported to increase circulating IGFBP2 during the early postoperative period, even before significant weight loss occurs (64, 65), a clinical trend conceptually summarized in Figure 1. Fahlbusch et al. observed lower serum IGFBP2 concentrations in obese men with NAFLD or steatohepatitis (NASH) compared to non-obese controls; notably, reduction of hepatic fat content through weight-loss intervention was associated with increased IGFBP2 levels (66). These clinical observations suggest that circulating IGFBP2 may reflect the metabolic state of insulin-sensitive tissues, particularly the liver and adipose tissue, although the underlying molecular pathways cannot be determined from observational studies alone.

Multi-omics analyses further support the potential of IGFBP2 as a biomarker for predicting T2DM and its complications, as well as obesity and NAFLD (67–70). Loesch et al. used polygenic risk scores (PGS) and identified significant genetic associations between IGFBP2 and T2DM/diabetic kidney disease (CKD) (71). Although Mendelian randomization (MR) analysis did not establish a significant causal effect of IGFBP2 on T2DM or CKD, mediation analysis revealed that IGFBP2 partially mediates the effects of T2D PGS on T2DM and CKD PGS on CKD (71). Subsequent survival analysis validated the association between IGFBP2 and the risk of cardiovascular and renal endpoint events in clinical trials, supporting its utility as a prognostic biomarker (71). Yang et al. employed machine learning models to assess the predictive capacity of early-pregnancy biomarkers for GDM and found that combining fasting plasma glucose (FPG) with IGFBP2 provided moderate discriminatory power (AUC = 0.80, accuracy = 0.72, sensitivity = 0.87, specificity = 0.57), suggesting that this combination could serve as an effective early prediction tool (72). A recent MR study reported that IGFBP2 has good diagnostic value for MASLD but found no significant causal relationship with disease risk (73). Thus, current human evidence supports IGFBP2 more strongly as a biomarker of metabolic dysfunction and metabolic responsiveness than as a confirmed causal driver of disease. Collectively, these findings highlight the value of IGFBP2 in predicting and stratifying metabolic disorders, while also suggesting that its clinical associations may offer mechanistic clues to the metabolic pathways disrupted in obesity, T2DM, and MASLD.

### 2.3 Nutritional factors influencing IGFBP2 expression and circulating levels

Nutritional status appears to be an important upstream determinant of IGFBP2 expression and circulating levels. Available evidence suggests that IGFBP2 is responsive not only to adiposity and insulin sensitivity, but also to changes in energy balance, dietary intervention, and weight-loss-associated metabolic remodeling (74, 75). In this regard, IGFBP2 may be viewed as a nutrition-sensitive metabolic signal linking dietary exposure to systemic metabolic adaptation.

One of the most consistent observations is that interventions producing negative energy balance or weight reduction are accompanied by increases in circulating IGFBP2. In a 1-year lifestyle modification program combining healthy eating counseling with physical activity and a daily energy deficit, circulating IGFBP2 increased by 43%, and the magnitude of the increase was closely associated with improvements in body fat distribution and lipoprotein-related parameters (75). Similarly, in obese patients undergoing bariatric surgery, circulating IGFBP2 rises early after surgery, even before major weight loss is achieved, suggesting that IGFBP2 responds rapidly to nutritional-metabolic improvement rather than simply reflecting later changes in body mass (65, 76). In line with this interpretation, bariatric-surgery-associated increases in IGFBP2 have been linked to improved insulin sensitivity, supporting the idea that nutritional interventions may modulate IGFBP2 in parallel with metabolic recovery. In adults with obesity-related fatty liver disease, lower serum IGFBP2 was observed in those with NAFLD or NASH, and reduction in liver fat after weight-loss intervention was accompanied by an increase in circulating IGFBP2 (66). In adipose tissue, obesity has also been associated with increased IGFBP2 DNA methylation and reduced IGFBP2 mRNA expression in visceral fat, together with lower circulating IGFBP2 (77), suggesting that nutritional excess may influence IGFBP2 partly through epigenetic remodeling in metabolically active tissues.

Current evidence for specific macronutrient effects is less developed. Some data suggest that broad dietary intervention and weight loss influence IGFBP2, but direct evidence isolating the independent effects of dietary carbohydrate, fat, or protein composition on circulating IGFBP2 remains limited. For example, a study in type 1 diabetes reported that a twofold difference in dietary protein intake did not materially alter the circulating IGF system, including IGFBP2, indicating that short-term manipulation of a single macronutrient may be insufficient to modify this axis in isolation (78). Likewise, small intervention studies with specific food-derived supplements such as lycopene or green tea have shown little clear effect on circulating IGFBP2 (79). By contrast, exploratory evidence suggests that time-restricted eating may alter circulating IGF-axis components, including IGFBP2, although this area remains preliminary and requires further confirmation (80).

Mechanistically, several pathways may explain why nutritional interventions influence IGFBP2. Leptin administration increases plasma IGFBP2 in ob/ob mice, indicating that hormonal signals related to energy sufficiency can regulate circulating IGFBP2, even though this effect does not appear to require intact hepatic leptin signaling (74). In humans, improvements in adiposity, liver fat, and insulin sensitivity are repeatedly accompanied by higher circulating IGFBP2 after dietary or surgical intervention, suggesting that IGFBP2 may integrate endocrine and tissue-level responses to nutritional remodeling (75, 76). Taken together, the available literature supports the view that IGFBP2 is influenced more consistently by overall nutritional-metabolic state and intervention-induced metabolic improvement than by any single dietary component currently studied in isolation. Future studies should clarify whether distinct dietary patterns or macronutrient distributions directly regulate IGFBP2 independent of weight loss and insulin sensitization.

## 3 Tissue-specific endocrine, autocrine, and paracrine roles of IGFBP2 in glucose and lipid metabolism

**FIGURE 2:**
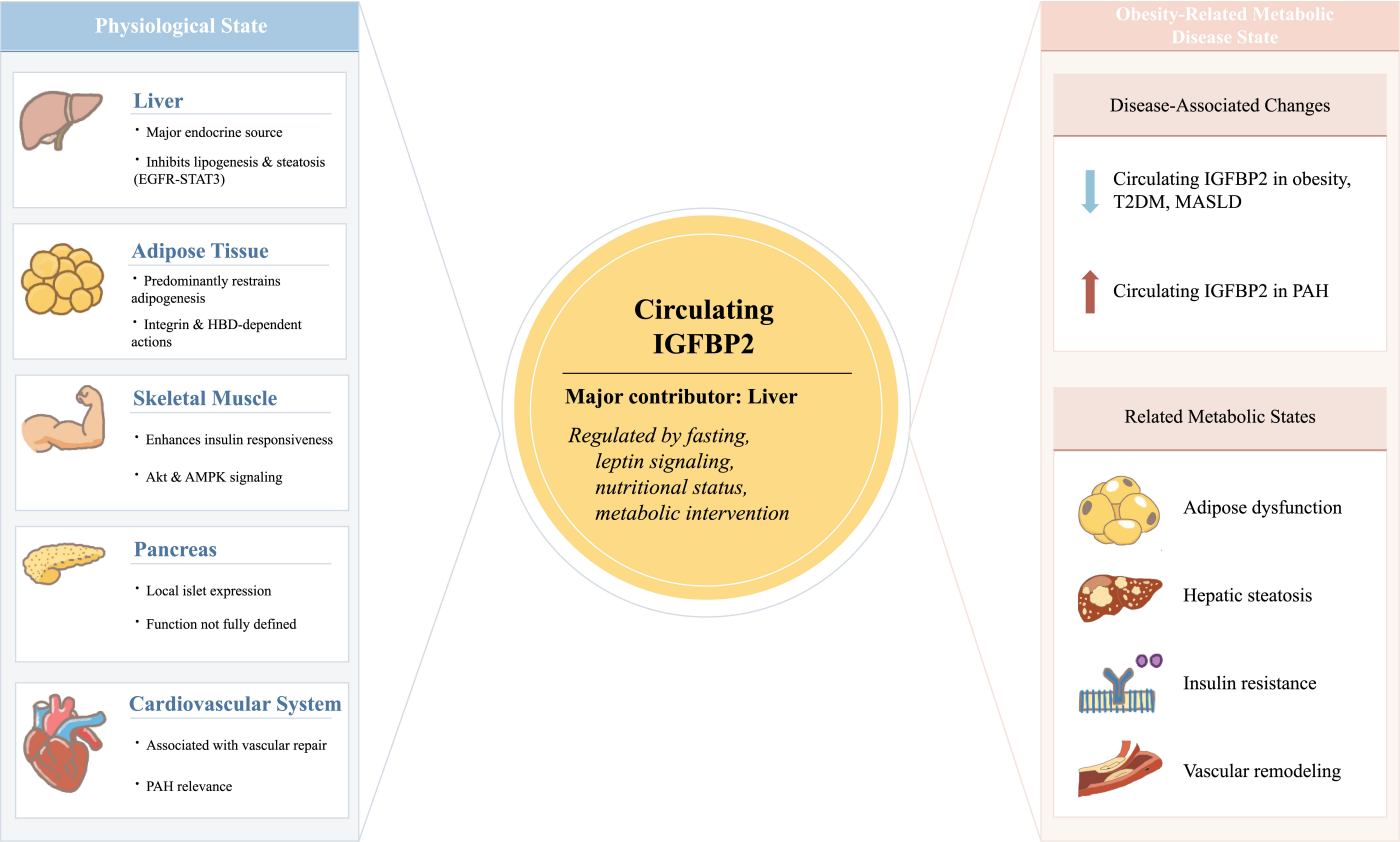
Tissue-specific and systemic actions of IGFBP2 in glucose and lipid metabolism under physiological and obesity-related metabolic disease states. IGFBP2 acts through both circulating endocrine and local tissue-associated mechanisms. The liver is considered a major contributor to the circulating IGFBP2 pool, whereas adipose tissue and skeletal muscle may also contribute to context-dependent local expression. In the liver, IGFBP2 suppresses lipogenesis and steatosis, partly through inhibition of EGFR–STAT3 signaling. In adipose tissue, IGFBP2 predominantly restrains adipogenesis in major experimental settings, although context-dependent pro-adipogenic effects have also been reported. In skeletal muscle, IGFBP2 supports insulin responsiveness and glucose uptake, whereas in the pancreas its local role remains incompletely defined. In the cardiovascular system, the clearest current evidence supports vascular repair-related actions and biomarker associations in pulmonary arterial hypertension, while direct myocardial metabolic mechanisms remain unresolved. In obesity-related metabolic disease states, circulating IGFBP2 is generally reduced in obesity, T2DM, and MASLD, whereas it may increase early after bariatric or weight-loss-associated metabolic intervention.

From an endocrine perspective, IGFBP2 acts through multiple modes across metabolic tissues. In some organs, particularly the liver, IGFBP2 functions both as a locally active factor and as a major contributor to the circulating pool (32, 81). In adipose tissue, IGFBP2 may exert local autocrine/paracrine effects while also contributing to systemic endocrine signaling in a context-dependent manner (47, 82). By contrast, in tissues such as the pancreas, skeletal muscle, and cardiovascular system, IGFBP2 is more often interpreted as a circulating metabolic signal, although local actions have also been proposed in specific settings (47, 83). Importantly, these modes of action are not mutually exclusive, and their relative importance likely varies with tissue type, metabolic state, and disease context. A schematic overview of these tissue-specific and systemic actions is provided in Figure 2, and the major tissue-specific modes of action, metabolic effects, and disease associations of IGFBP2 are summarized in Table 1.

**TABLE 1:** Tissue-specific metabolic actions, signaling mechanisms, and disease relevance of IGFBP2.

Tissue/context	Predominant mode of action	Change in obesity-related metabolic disease	Major metabolic effects	Key signaling pathways/molecular mechanisms	Disease association/biomarker implication	Representative references
Liver	Autocrine and endocrine (major contributor to the circulating pool)	Generally reduced in obesity, insulin resistance, and steatotic liver disease	Suppresses hepatic lipid deposition and lipogenesis; limits steatosis; supports liver homeostasis and regeneration	Direct binding to EGFR and suppression of EGFR–STAT3 signaling; transcriptional regulation involving PPARγ2–RXRα; altered DNA methylation contributes to dysregulation	Strong evidence supports an inverse association with MASLD/hepatic steatosis; promising biomarker and mechanistically one of the best-supported tissues in this review	(32, 51, 74, 81, 84–86)
Pancreas/islet microenvironment	Mainly local (endocrine-relevant and possible paracrine/autocrine-like actions), with probable modulation by circulating IGFBP2	Pancreas-specific expression dynamics remain insufficiently defined; circulating IGFBP2 is generally lower in insulin-resistant states	Possible involvement in islet microenvironment regulation; direct effects on β-cell function remain incompletely defined	Pancreatic IGFBP2 is expressed in islets/acinar tissue; may interact with extracellular matrix, proteoglycans, and integrin-related pathways, but no definitive pancreas-specific signaling mechanism has been established	Currently more relevant to pathophysiologic interpretation than to a validated pancreas-specific biomarker claim; should be discussed cautiously	(34, 87–91)
Adipose tissue	Autocrine/paracrine and endocrine	Generally lower circulating IGFBP2 in obesity; local adipose expression is context-dependent	Usually restrains adipose expansion and adipogenesis in dominant experimental settings; may improve insulin sensitivity and glucose handling; pro-adipogenic effects reported in specific models	Anti-adipogenic actions linked to HBD-dependent effects and integrin–ERK1/2 signaling; pro-adipogenic effects reported via JNK/Akt in specific MSC contexts	Supported mainly as a risk-associated biomarker for obesity-related metabolic dysfunction; mechanistic effects are context-dependent across depots and cell types	(49, 60, 77, 92, 94–97)
Skeletal muscle	Mainly endocrine, with possible local actions in the muscle microenvironment	Tissue-specific expression trend remains incompletely defined; functionally, lower IGFBP2 is associated with reduced insulin sensitivity	Enhances insulin responsiveness and glucose uptake; may influence myogenic remodeling and muscle phenotype adaptation	Linked to Akt phosphorylation in differentiated myotubes; possible involvement of AMPK-related pathways, although direct muscle-specific evidence remains limited	Best regarded as part of the systemic insulin-sensitivity signature of IGFBP2 rather than as a standalone skeletal-muscle biomarker	(27, 98–103)
Circulating IGFBP2/systemic metabolic state	Endocrine (systemic biomarker perspective)	Circulating IGFBP2 is generally reduced in obesity, T2DM, and MASLD; rises early after bariatric surgery and weight-loss-associated metabolic improvement	Associated with lower future risk of T2DM, better insulin sensitivity, lower adiposity burden, and improved metabolic responsiveness after intervention	Reflects integrated signaling across multiple organs rather than a single pathway; influenced by nutritional status, weight loss, and broader metabolic remodeling	The strongest current human evidence supports predictive/risk-stratification biomarker use in obesity, T2DM, and MASLD; causality remains incompletely established	(34, 62–66, 71–80)
Cardiovascular system/vasculature	Mainly endocrine and local vascular actions; highly context-dependent	No single uniform direction; circulating levels are elevated in PAH, whereas relationships with atherosclerotic phenotypes are heterogeneous	Possible roles in endothelial repair and pulmonary vascular remodeling; direct metabolic effects in the heart remain insufficiently defined	Integrin α5β1-mediated endothelial progenitor cell adhesion is the clearest local mechanism; myocardial metabolic mechanisms remain unresolved	Circulating IGFBP2 has biomarker potential in PAH; associations with atherosclerotic vascular phenotypes are complex, population-dependent, and not yet mechanistically resolved	(38, 104–121)

The predominant mode of action and disease-associated expression patterns of IGFBP2 are context-dependent and may vary by tissue type, metabolic state, and study design. Evidence is strongest for the liver, whereas pancreas- and cardiovascular-specific mechanistic evidence remains comparatively limited. IGFBP2, insulin-like growth factor-binding protein 2; T2DM, type 2 diabetes mellitus; MASLD, metabolic dysfunction-associated steatotic liver disease; EGFR, epidermal growth factor receptor; STAT3, signal transducer and activator of transcription 3; RXRα, retinoid X receptor alpha; HBD, heparin-binding domain; MSCs, mesenchymal stem cells; AMPK, AMP-activated protein kinase; PAH, pulmonary arterial hypertension. Akt, protein kinase B; ERK, extracellular signal-regulated kinases; JNK, c-Jun N-terminal kinase; PPAR, peroxisome proliferators-activated receptors.

### 3.1 Autocrine and endocrine roles of IGFBP2 in the liver

In the liver, IGFBP2 functions both as a locally active hepatoprotective factor and as a major contributor to the circulating pool. The liver is a primary site of IGFBP2 synthesis (37), releasing it into the circulation through endocrine mechanisms, while hepatic IGFBP2 also appears to exert local metabolic effects within hepatocytes (84). Transcriptional regulation of IGFBP2 involves peroxisome proliferators-activated receptor γ2 (PPARγ2)–retinoid X receptor α (RXRα) binding to a peroxisome proliferator response element site in its promoter; this interaction is diminished in morbid obesity with high insulin resistance, leading to reduced IGFBP2 expression (85). In addition, altered DNA methylation of IGFBP2 contributes to hepatic lipid accumulation (81), indicating that both transcriptional and epigenetic mechanisms participate in the hepatic dysregulation of IGFBP2.

Functionally, IGFBP2 suppresses lipid deposition and lipogenesis in hepatocytes. In hepatocytes and steatotic liver models, restoration or induction of IGFBP2 is associated with reduced lipid accumulation and lipogenic gene expression (51, 86). Leptin signaling has also been reported to stimulate hepatic IGFBP2 expression and may mediate part of leptin’s metabolic effects (74). Consistent with this, liver-specific IGFBP2 deficiency exacerbates hepatic steatosis and is associated with increased hepatic triglycerides, total cholesterol, and free fatty acids, supporting a direct role for hepatic IGFBP2 in maintaining lipid homeostasis (32). Together, these findings suggest that reduced hepatic IGFBP2 in obesity and insulin-resistant states may contribute not only to lower circulating IGFBP2 levels but also to the progression of steatotic liver disease.

Recent studies have further suggested that IGFBP2 participates in liver homeostasis and regeneration (84). IGFBP2-expressing hepatocytes in zone 2 contribute to liver repopulation during normal homeostasis and after injury, and fasting alters liver zonation in a manner associated with IGFBP2-positive cells (84). Although these observations extend beyond classical glucose and lipid metabolism, they support the broader view that hepatic IGFBP2 is an active regulator of metabolic adaptation rather than merely a circulating binding protein.

### 3.2 Endocrine-relevant and local actions of IGFBP2 in the pancreas

In the pancreas, available evidence indicates that IGFBP2 is expressed in pancreatic tissue and may participate in local islet signaling, although its specific role remains far less well defined than in the liver. Developmental studies have detected IGFBP2 mRNA in both pancreatic islets and acinar tissue (87), and β-cell/islet models have further suggested that pancreatic cells can produce or release IGFBP-2 (88), supporting the possibility of local actions within the islet microenvironment.

Evidence directly linking pancreatic IGFBP2 to β-cell function remains limited and context-dependent. In a fetal ovine model of intrauterine growth restriction, IGFBP2 expression was increased in the pancreas and isolated islets, whereas insulin mRNA was reduced, suggesting that pancreatic IGFBP2 is altered under endocrine stress, although this model does not establish a definitive signaling mechanism for IGFBP2 in β-cells (89).

At the clinical level, circulating IGFBP2 is more consistently associated with systemic insulin sensitivity than with a specific pancreatic mechanism. Higher serum IGFBP2 levels have been linked to better insulin sensitivity and a more favorable metabolic profile in humans (34, 90). However, current evidence does not support the pancreas as a major physiological source of circulating IGFBP2; rather, it is more likely to represent a target tissue of circulating IGFBP2, while any local pancreatic role remains unclear. IGFBP2 is known to interact with extracellular matrix components, cell-surface proteoglycans, and integrin-related pathways, which may influence local IGF availability and signaling (91). Although these interactions have not been clearly defined in pancreatic tissue, they suggest that IGFBP2 may affect the islet microenvironment. Taken together, the pancreatic literature remains limited and largely descriptive, and a definitive mechanism has yet to be established.

### 3.3 Autocrine/paracrine and endocrine roles of IGFBP2 in adipose tissue

In adipose tissue, IGFBP2 may contribute to systemic endocrine signaling while also acting locally through autocrine/paracrine mechanisms, particularly within specialized adipose microenvironments such as bone marrow beige fat (92). Serum IGFBP2 levels are significantly lower in obese individuals than in those with normal weight (77, 93). Consistently, transgenic overexpression of IGFBP2 in rodents protects against both diet-induced and age-related obesity (49), and mice with adipocyte-specific overexpression of IGFBP2 are resistant to obesity and insulin resistance even under overfeeding conditions (94). In obese children, IGFBP2 is expressed in abdominal subcutaneous adipocytes, and its expression increases with the degree of obesity independently of insulin sensitivity, suggesting that locally expressed IGFBP2 may act as an autocrine/paracrine factor that restrains further adipose expansion (60).

The role of IGFBP2 in adipocyte differentiation, however, remains controversial. Xi et al. (95) reported that IGFBP2 inhibits preadipocyte differentiation and downregulates key adipogenic markers including PPARγ, adipocyte protein 2, and adiponectin. Using an *IGFBP2^–/–^*mouse model, the same group further showed that subcutaneous administration of PEGylated HBD peptides reduced total fat mass, with HBD2 producing a marked reduction in both inguinal and visceral fat, whereas HBD1 predominantly reduced visceral fat accumulation (95). These findings support an anti-adipogenic role of IGFBP2-related signaling *in vivo*. Similarly, Ferrero et al. identified a population of mesothelial-like stromal cells in human omental adipose tissue that highly express IGFBP2 (96). Secreted IGFBP2 from these cells inhibited the adipogenic differentiation of neighboring human adipose stem/precursor cells (hASPCs) through activation of integrin–extracellular signal-regulated kinase1/2 signaling, and this effect was reversed by IGFBP2 knockdown or integrin blockade (96). Together, these studies suggest that in primary adipose tissue, particularly within specialized stromal niches, IGFBP2 more often acts to limit adipogenesis.

In contrast, pro-adipogenic effects of IGFBP2 have also been reported in specific experimental settings. Wang et al. showed that IGFBP2 overexpression enhanced c-Jun N-terminal kinase (JNK) and protein kinase B (Akt) phosphorylation in mesenchymal stem cells (MSCs), thereby promoting adipogenic differentiation (97). In particular, IGFBP2-overexpressing umbilical cord Wharton’s jelly MSCs (WJCMSCs) displayed increased lipid deposition after adipogenic induction, suggesting that IGFBP2 may enhance adipocyte formation under certain cellular contexts (97). Therefore, the apparently divergent effects of IGFBP2 on adipogenesis likely reflect differences in cellular origin, adipose depot, microenvironmental cues, developmental stage, and dominant downstream signaling pathways. Overall, the available evidence favors an inhibitory role of IGFBP2 in primary adipose tissue biology, whereas pro-adipogenic effects appear to be more model- and context-specific.

### 3.4 Endocrine and local actions of IGFBP2 in skeletal muscle

In skeletal muscle, IGFBP2 is more often discussed as a circulating metabolic signal, although local actions in the muscle microenvironment have also been reported. Experimental evidence indicates that IGFBP2 can influence myoblast biology by promoting proliferation while restraining terminal differentiation (98, 99). In C2C12 myoblasts, reduced early availability of IGFBP2 impaired subsequent differentiation and altered myotube hypertrophy-related responses, suggesting that IGFBP2 contributes to the coordination of myogenic progression (98). Consistent with this, Wang et al. showed that IGFBP2 overexpression facilitated myoblast proliferation and hampered myotube formation, with reduced myogenic differentiation marked by altered MyHC expression (99).

In differentiated skeletal muscle cells, the evidence for IGFBP2 relates to glucose metabolism and insulin responsiveness. In cultured human skeletal myotubes, leptin directly increased IGFBP2 expression through signal transducer and activator of transcription 3 (STAT3)- and phosphatidyqinositol-3 kinase (PI3K)-dependent signaling, whereas silencing IGFBP2 reduced both leptin- and insulin-stimulated Akt phosphorylation and impaired glucose uptake (27). This study suggests that IGFBP2 contributes to insulin-sensitive glucose metabolism in skeletal muscle. Evidence from *in vitro* metabolic studies further suggests that IGFBP2-associated glucose uptake may involve PI3K/Akt- and AMPK-related pathways, although the precise muscle-intrinsic signaling mechanism remains incompletely defined (100, 101).

Beyond glucose handling, IGFBP2 has also been linked to skeletal muscle remodeling and wasting states. Muscle-specific overexpression of IGFBP2 has been reported to promote a slower muscle phenotype in dystrophin-deficient mice, indicating that IGFBP2 may influence muscle functional adaptation under pathological conditions (102). In addition, higher circulating IGFBP2 levels have been associated with muscle wasting and malnutrition in cancer cachexia-related settings, suggesting potential biomarker relevance in muscle loss conditions rather than a defined causal mechanism (103). Although physical activity can alter circulating IGFBP2 levels, direct evidence that IGFBP2 mediates exercise-induced skeletal muscle metabolic remodeling remains limited. Overall, current evidence suggests that IGFBP2 supports skeletal muscle metabolic homeostasis primarily by preserving insulin responsiveness and influencing myogenic remodeling, whereas many of its muscle-specific mechanisms remain to be clarified.

### 3.5 Context-dependent endocrine and local actions of IGFBP2 in the cardiovascular system

In the cardiovascular system, IGFBP2 is more often interpreted as a circulating endocrine signal acting on cardiovascular tissues. Compared with the relatively better-characterized roles of IGFBP2 in the liver, adipose tissue, and skeletal muscle, its direct metabolic functions in the heart and vasculature remain less clearly defined. Current evidence suggests that IGFBP2 may participate in endothelial repair and pulmonary vascular remodeling but whether these actions are predominantly protective or pathogenic likely depends on the specific disease context and cell type involved (38, 104–107).

In the heart, direct mechanistic evidence linking IGFBP2 to myocardial metabolism remains scarce. At present, it is unclear whether IGFBP2 directly affects cardiomyocyte glucose utilization, fatty acid oxidation, or metabolic flexibility, and its potential role in myocardial injury responses requires further investigation.

The arterial wall comprises multiple cell types, including endothelial cells and smooth muscle cells (104). Pulmonary arterial hypertension (PAH) is characterized by progressive remodeling and increased resistance of small pulmonary arteries, driven primarily by abnormal proliferation and migration of vascular wall cells. Recent clinical studies have reported significantly elevated circulating IGFBP2 levels in PAH patients compared with controls, with higher levels associated with greater disease severity and worse outcomes (105–107). In vascular endothelial progenitor cells, IGFBP2 interacts with integrin α5β1 via its RGD motif, enhancing adhesion to endothelial cells and supporting a potential role in vascular repair-related processes (38).

The involvement of IGFBP2 in the initiation and progression of atherosclerosis remains incompletely defined and may involve multiple mechanisms. Atherosclerosis is characterized by lipid accumulation within the arterial intima and disordered lipid metabolism (104, 108). Circulating monocytes transmigrate across the endothelium into the intima, where they engulf modified lipoproteins and differentiate into lipid-laden macrophages or foam cells (109, 110). Atherosclerotic lesions also comprise dysfunctional endothelial cells, vascular smooth muscle cells, and T cells (104, 111–113). Against this pathophysiological background, lower circulating IGFBP2 levels have been associated with adverse cardiometabolic phenotypes and dyslipidemia-related traits, suggesting that reduced IGFBP2 may accompany a more pro-atherogenic metabolic milieu (33, 114–116), However, direct interventional evidence does not uniformly support a protective role, as intravenous IGFBP2 administration aggravated coronary atherosclerosis and enhanced inflammatory responses in hypercholesterolemic rabbits (117). Human observational studies further suggest that the relationship between IGFBP2 and atherosclerotic vascular phenotypes is complex and may vary across populations and analytical models (118–121).

Collectively, these findings suggest that IGFBP2 has context-dependent associations with cardiovascular disease. Its circulating levels appear to have biomarker potential in PAH and broader cardiometabolic risk states (105–107, 115), whereas local vascular actions, such as integrin-dependent effects on endothelial progenitor cells, point to possible roles in vascular remodeling or repair (38). Nevertheless, its direct mechanistic role in myocardial metabolism and atherosclerotic progression remains insufficiently resolved.

## 4 Conclusions and future directions

Research over the past decade has established IGFBP2 as a pleiotropic regulator modulating glucose and lipid homeostasis in a tissue-specific and context-dependent manner. This review highlights the intricate signaling networks through which IGFBP2 acts across metabolic tissues, including EGFR-STAT3 signaling in the liver, integrin-dependent pathways in adipose and vascular contexts, and insulin-responsive signaling in skeletal muscle, with possible additional links to AMPK-related mechanisms. In addition, emerging evidence suggests that IGFBP2 is responsive to nutritional status, weight-loss interventions, and broader metabolic remodeling, supporting its role as a nutrition-sensitive component of metabolic regulation. However, its effects, particularly in adipose tissue and the cardiovascular system, remain highly context-dependent, ranging from metabolically protective associations in some settings to potential links with pathological remodeling or adverse vascular phenotypes in others. A major challenge lies in deciphering the molecular determinants, including post-translational modifications and tissue microenvironmental cues, that govern these divergent outcomes.

Clinical studies consistently associate lower circulating IGFBP2 levels with increased risk and severity of obesity, T2DM, and MASLD. Its rapid increase following interventions such as bariatric surgery, even before substantial weight loss, further supports its close relationship with metabolic status and responsiveness to intervention. Multi-omics analyses and emerging machine learning approaches further bolster its potential utility as a biomarker for predicting these metabolic disorders and their complications. However, the well-established oncogenic role of IGFBP2 in several cancer types introduces considerable complexity for its diagnostic and especially therapeutic application in the metabolic realm. Future biomarker strategies may need to exploit specific post-translationally modified forms or tissue-specific measurements. Future research might prioritize several key aspects: (i) Elucidating the precise IGFBP2-binding receptors and downstream signaling cascades in understudied contexts. (ii) Defining how the metabolic and nutritional microenvironment, extracellular matrix composition, epigenetics, and coexisting pathologies dictate IGFBP2’s cellular actions. (iii) determining whether distinct dietary patterns or macronutrient distributions regulate IGFBP2 independently of weight loss and insulin sensitization; and (iv) Developing strategies, potentially leveraging its distinct functional domains, for tissue-specific IGFBP2 modulation that enhances beneficial metabolic effects without activating oncogenic pathways, while rigorously validating IGFBP2-related biomarkers across diverse populations and stages of metabolic disease progression.
